# Whole Genome Sequence Analysis of Porcine Astroviruses Reveals Novel Genetically Diverse Strains Circulating in East African Smallholder Pig Farms

**DOI:** 10.3390/v12111262

**Published:** 2020-11-05

**Authors:** Joshua O. Amimo, Eunice M. Machuka, Edward O. Abworo, Anastasia N. Vlasova, Roger Pelle

**Affiliations:** 1 Department of Animal Production, Faculty of Veterinary Medicine, University of Nairobi, P.O. Box 29053, Nairobi 00625, Kenya; 2 Food Animal Health Research Program, Department of Veterinary Preventive Medicine, The Ohio State University, 1168 Madison Avenue, Wooster, OH 44961, USA; vlasova.1@osu.edu; 3 Biosciences Eastern and Central Africa-International Livestock Research Institute (BecA-ILRI) Hub, P.O. Box 30709, Nairobi 00100, Kenya; E.Machuka@cgiar.org (E.M.M.); e.okoth@cgiar.org (E.O.A.); r.pelle@cgiar.org (R.P.)

**Keywords:** porcine astroviruses, linear antigenic epitopes, recombination, glycosylation, whole genome sequences, East Africa

## Abstract

Astroviruses (AstVs) are widely distributed and are associated with gastroenteritis in human and animals. The knowledge of the genetic diversity and epidemiology of AstVs in Africa is limited. This study aimed to characterize astroviruses in asymptomatic smallholder piglets in Kenya and Uganda. Twenty-four samples were randomly selected from a total of 446 piglets aged below 6 months that were initially collected for rotavirus study and sequenced for whole genome analysis. Thirteen (13/24) samples had contigs with high identity to genus *Mamastrovirus*. Analysis of seven strains with complete (or near complete) AstV genome revealed variable nucleotide and amino acid sequence identities with known porcine astrovirus (PoAstV) strains. The U083 and K321 strains had nucleotide sequence identities ranging from 66.4 to 75.4% with the known PoAstV2 strains; U460 strain had nucleotide sequence identities of 57.0 to 65.1% regarding the known PoAstV3; and K062, K366, K451, and K456 strains had nucleotide sequence identities of 63.5 to 80% with the known PoAstV4 strains. The low sequence identities (<90%) indicate that novel genotypes of PoAstVs are circulating in the study area. Recombination analysis using whole genomes revealed evidence of multiple recombination events in PoAstV4, suggesting that recombination might have contributed to the observed genetic diversity. Linear antigen epitope prediction and a comparative analysis of capsid protein of our field strains identified potential candidate epitopes that could help in the design of immuno-diagnostic tools and a subunit vaccine. These findings provide new insights into the molecular epidemiology of porcine astroviruses in East Africa.

## 1. Introduction

Porcine astroviruses (PoAstVs) belong to the family *Astroviridae* consisting of two genera, *Avastrovirus* and *Mamastrovirus*, based on the host ranges of avian and mammalian species, respectively [1]. Astroviruses (AstVs) have been isolated from fecal samples of a wide variety of mammals and birds [1,2,3,4]. AstVs are known to be the second most prevalent cause (after rotaviruses) of viral gastroenteritis in infants and children [5], and they have been reported in water sources and sewage samples [6,7]. The first AstVs were identified by electron microscopy in 1975 in Scotland in fecal samples from infants hospitalized with diarrhea [8]. AstVs spread via the fecal–oral route, through direct contact, or via contaminated food and water [9]. The number of reports on AstV detection is relatively low, and only one exists for East African children [10]. Rapid mutation of AstVs, and co-infection of multiple AstV strains in a single individual [3,11], could result in a recombination event from which a zoonotic strain may emerge. Emergence of porcine–human AstV recombinants has been reported in regions where pigs and humans live closely, such as our study region, with suspected human to pig transmission, although the reverse has not been reported [12].

AstVs are small, nonenveloped viruses each containing a positive-sense single-stranded RNA of 6.4–7.3 kb in size. The AstV genome consists of three open reading frames (ORFs), untranslated regions (UTR) at both 5′ and 3′ ends, and a poly-A tail at the 3′ end. The genome has a frame shift structure between ORF1a and ORF1b [1,13]. The three ORFs encode for nonstructural protease (ORF1a), RNA-dependent RNA polymerase (ORF1b), and structural capsid protein (ORF2), respectively [11]. Genome analysis has shown that ORF1b is the most conserved while ORF2 is highly divergent due to selective pressure in this region. Recently, the Astroviruses Study Group, International Committee on Taxonomy of Viruses (ICTV) (http://ictvonline.org/virusTaxonomy.asp), proposed a classification based on the amino acid sequence of ORF2 which encodes the capsid polyprotein and represents the most variable region of the genome [12,14]. Currently, there are 19 *Mamastrovirus* species (MAstV 1–19) recognized by the ICTV. In pigs, the first porcine astrovirus (PoAstV) was identified by electron microscopy in 1980 [15]. To date, four more PoAstV types (PoAstV2–PoAstV5) have been identified [16,17,18,19]. The ICTV has classified PoAstV1 within *Mamastrovirus* 3; classification of the other four porcine astroviruses (PoAstV2–PoAstV5) as other astroviruses species has not been confirmed [20]. Association of PoAstV with gastroenteritis has been reported [15,17,21,22]; however, PoAstVs have also been found in healthy pigs [19,23,24,25,26,27]. Astroviruses are among the least studied enteric viruses, despite their clinical importance [28], probably due to lack of well-established animal models and/or fewer full genome sequences available. Additionally, there are limited established culture systems for propagating AstVs [22,29]. Studies of genetic and antigenic diversity among PoAstV strains in a given location present a challenge for the development of accurate diagnostic assays and vaccines, and hence improved disease prevention [24]. Currently, there are no PoAstV vaccines available commercially. In this study, the complete (or near complete) genomes of seven porcine astroviruses were analyzed and characterized, which revealed novel porcine astrovirus strains. Additionally, we determined a potential linear antigenic epitope on the capsid region of our PoAstV strains with predicted high antigenicity, which could be useful in the design of immuno-diagnostic tools and subunit vaccines.

## 2. Materials and Methods

### 2.1. Ethics Statement

This study was conducted according to the ethical guidelines of both the Commonwealth Scientific Research and Industrial Organization (CSIRO)—Social Science Human Research Ethics Committee (CSSHREC), Australia (approval 059/11), and the Institutional Animal Care and Use Committee (IACUC) of International Livestock Research Institute (ILRI), Kenya (Ref: 2011-04).

### 2.2. Selection of Samples

Twenty four (24) samples were randomly selected from a total of 446 piglets aged below 6 months that were initially collected to study rotaviruses distribution and diversity in western Kenya and eastern Uganda [30]. The sample selection was based on the initial rotavirus prescreening.

### 2.3. Processing of Fecal Samples, RNA Library Preparation, and Sequencing

The sample and library preparation and sequencing of samples used in this study have been described in detail by Amimo et al., 2016 [2]. Briefly, fecal suspensions were filtered using 0.22 μm membrane filters to remove eukaryotic and bacterial cell debris. Fecal filtrate was treated with DNases and RNases (Takara, Japan) and incubated for 2 h at 37 °C to destroy free unprotected nucleic acids. Extraction of viral RNA was performed using the QIAamp Viral RNA Mini Kit (Qiagen, USA) according to the manufacturer’s instructions. Total RNA was used in library construction using the TruSeq™ RNA library preparation kit v2 (Illumina^®^, USA) according to the manufacturer’s instructions. The final size and concentration of each library were estimated using a Bioanalyzer (Agilent, Santa Clara, CA, USA) and the Qubit, respectively. A ten (10 nm) library pool was prepared by mixing the 24 samples to achieve an equal molar concentration for each library. Pooled libraries were sequenced by the MiSeq (Illumina^®^) platform using sequencing runs of 2 × 150 paired-end reads at the BecA-ILRI Hub genomics laboratory facility in Nairobi, Kenya.

### 2.4. Data Analysis

Comprehensive sequence data quality check, de novo contig assembly, and contig classification have been described by Amimo et al., 2016 [2]. Briefly, 27,260,153 raw reads were processed by trimming the adapters based on Phred quality scores, followed by removal of duplicate reads. Reads that passed quality check were used for de novo contig assembly using Trinity software by applying criteria of >50% overlap and >90 % overlap identity [31]. Assembled contigs (185,827) were classified by BLASTx search against the NCBI non-redundant protein database (NR), with an e-value cutoff of 10^−10^. The contigs with significant BLASTx hits were additionally classified using a second BLASTx search with an e-value cutoff of 10^−4^. Of the 185,827 contigs assembled, 98.8% (183,532) matched the sequences in the GenBank while 1.2% were unassigned. Using preliminary results, the contigs from 13 samples, having hits with high similarity to astroviruses, were analyzed with the Metavir2 beta version, the Web-based tool for virome analysis [32]; the taxonomic composition was computed from a BLAST comparison with the Refseq complete viral genomes protein sequences database from NCBI using BLASTp and threshold of 10^−3^ on the e-value. Additionally, to confirm that the 7 complete genomes of astroviruses from the seven individual samples were true astrovirus genomes, all the reads in a respective sample that were classified belonging to the *Astroviridae* family were aligned to long contigs using BWA. The contig quality was further checked based on this alignment to ensure all positions of the consensus contigs were filled with consensus nucleotide for the position.

#### 2.4.1. Phylogenetic Analysis

Multiple sequence alignment was performed using Clustal Omega (ClustalO) Web server (http://www.ebi.ac.uk/Tools/msa/clustalo/) to analyze the percentage identity with other genomes at nucleotide and amino acid levels. Phylogenetic analyses of assembled genomes and predicted proteins were performed in MEGA X [33] after building alignment by ClustalW algorithm and subsequent tree generation using the neighbor-joining method [34] with 1000 bootstrap replicates.

#### 2.4.2. Recombination Analysis

A nucleotide alignment was created by using ClustalW on the full genome sequences of 28 mamastroviruses, including the seven porcine astroviruses from this study and 21 known astroviruses from swine, bovines, camels, and humans. The representative whole genome sequences of the known astroviruses from the NCBI GenBank were selected based on the initial BLASTn search results of each of our seven field strains and phylogenetic analysis. Potential recombination patterns were screened by using RDP, GENECONV, MaxChi, Chimaera, SiScan, 3Seq, and Bootscan in the Recombination Detection Program (RDP4, Version 4.94) while following the instruction manual [35], using the step-down correction for multiple comparisons and a *p*-value cutoff of 0.01. Regions of potential recombinant interest were also checked by the above methods. Recombination events were considered only when involving at least one of our 7 newly generated sequences and when supported by highest acceptable *p*-value of 0.05 with all of the above methods.

#### 2.4.3. Linear Antigen Epitope Prediction

SVMTriP Web-based software [36], which predict linear antigen epitopes based on a support vector machine to integrate tri-peptide similarity and propensity, was used to predict potential linear antigen epitopes with the capsid protein sequences of our PoAstVs. The astrovirus capsid gene is responsible for viral attachment and entry into host cells; therefore, realistic prediction of antigenic epitopes could help with the design of vaccine components and immuno-diagnostic reagents. The epitopes predicted by SVMTrip software were further analyzed with the IEDB analysis resource and the immuno-medicine group tool—Web-based programs which predict segments from within a protein sequence that are likely to be antigenic via eliciting antibody responses [37,38]. Antigenicity of predicted candidate epitopes was determined using VaxiJen v2.0, protective antigen, tumor antigens, and subunit vaccines prediction server [39]. This server uses auto cross covariance (ACC) transformation of selected protein sequences based on unique amino acid properties. Each sequence is used to find out 100 known antigens and 100 non-antigens. The identified sequences are tested for antigenicity by leave-one-out cross-validation and overall external validation. The prediction accuracy is up to 89%. Thereafter, Swiss model was used for modelling the 3D structures of capsid proteins, as it is fully automated and provides complete stoichiometry and the overall structures of the complexes as inferred by homology modelling [40]. Conformational B-cell epitopes from the 3D models of detected PoAstV proteins were predicted by ElliPro, a Web tool designed by Thornton’s method together with MODELLER program of a residue clustering algorithm and Jmol viewer [41].

#### 2.4.4. Glycosylation Analysis

Glycosylation is an important post-translational modification, and is known to influence protein folding, localization, trafficking, and solubility; antigenicity; biological activity and half-life; and cell–cell interactions. We investigated the spread of known and predicted N-glycosylation sites across the capsid protein of the 7 PoAstV field strains identified in this study using NetNglyc software [42]. This software uses artificial neural networks that examine the sequence context of Asn–X–Ser/Thr sequons (Asn = asparagine, Ser = serine, Thr = threonine, and X = any other amino acid except proline) and distinguishes glycosylated sequences from non-glycosylated ones. The predictions are only shown on Asn–X–Ser/Thr sequences, since only asparagine residues within Asn–X–Ser/Thr (and in some cases, Asn–X–Cys) are N-glycosylated in-vivo.

## 3. Results and Discussion

### 3.1. General Features of Complete Genome sequences of East African Porcine Astroviruses

The lengths of RNA genome sequences of the seven newly identified field strains (U083, K321, U460, K456, K451, K366, K062), excluding the 30 adenines [poly(A) tail)] at the 3′ end, varied from 5281 to 6649 nt, as shown in Table 1. They had typical AstV genomic organization with the three predicted main ORFs (ORF1a, ORF1b, and ORF2) preceded by a 5’ untranslated region (UTR) and ending with a 3’ UTR, with ribosomal slippage site between ORF1a and ORF1b. The 5’ UTR regions of K451 and U460 strains were not assembled. A frameshift heptamer, AAAAAAC, followed by a stem-loop structure, was present near the 3′ end of ORF1a in all the strains, which is signal for a ribosomal frameshift during translation to generate the replicase polyprotein ORF1ab [43]. PoAstVs detected in this study showed the conserved tyrosine residue within the TEEEY motif in the viral protein genome-linked (VPg) putative protein at the 3′ end of ORF1a (PoAstV3 contained SEEEY). Additionally, a typical YGDD motif was conserved in the middle of the predicted RNA-dependent RNA polymerase (RdRp) protein of all our strains [43,44,45]. All the strains also contained a conserved sequence located at the junction of RdRp and capsid region (UUUGGAGGGG(A/C)GGACCAAA(G/A)_8/11_AUGGC), which was proposed to be a regulatory element used as a promoter for sgRNA transcription [14,43]. Finally, all our strains contained a trypsin-like peptidase domain in the nonstructural protein 1a and an astrovirus capsid protein precursor domain.

### 3.2. Genetic Diversity and Phylogenetic Analysis

**Figure 1:**
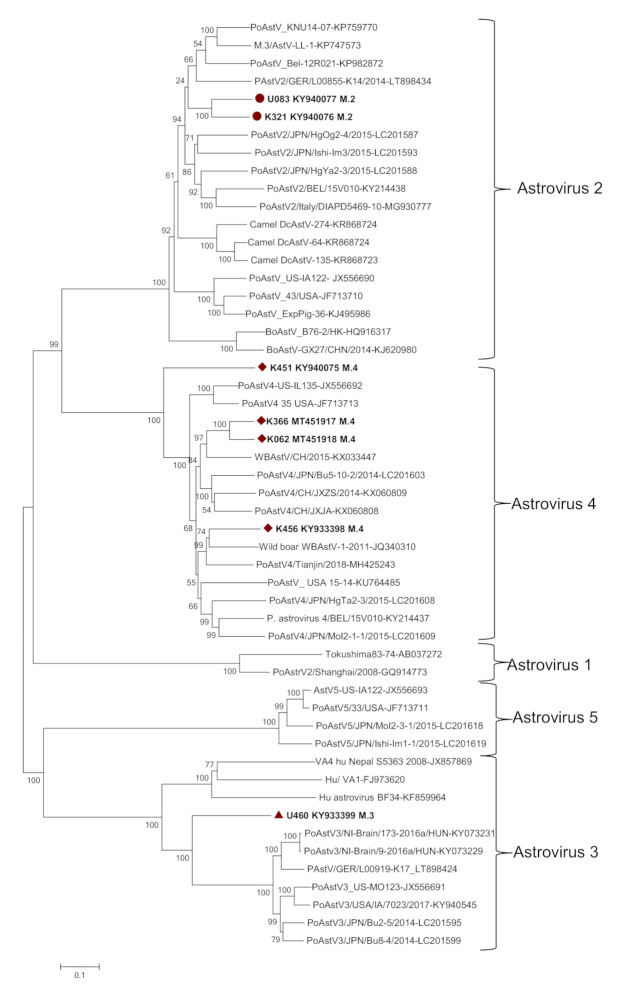
Phylogenetic analysis based on the complete (nearly complete, U460) nucleotide sequences of the full-length genome of East African PoAstVs (**bold**) and representative AstVs of humans and other species in the GenBank. Multiple sequence alignments were performed using the ClustalO program. The evolutionary history was inferred by using the maximum likelihood method based on the general time reversible model in MEGA X [33]. The scale bar is given in numbers of substitutions per site. Phylogeny was inferred following 1000 bootstrap replications, and the node values show percentage bootstrap support.

**Figure 2:**
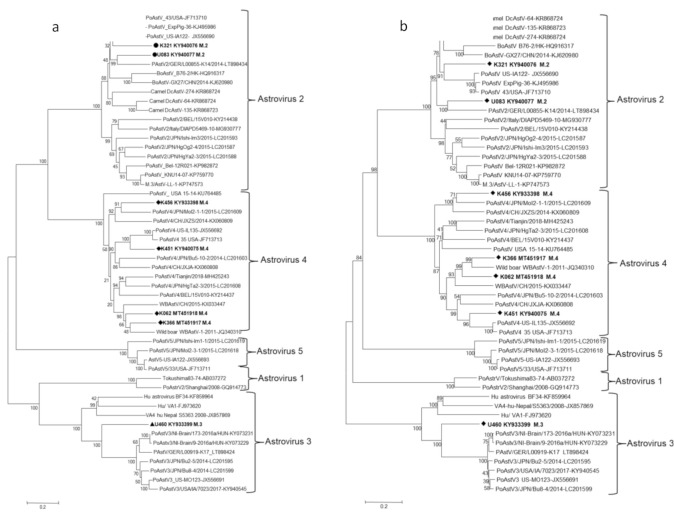
Phylogenetic tree based on the nucleotide (**a**) and amino acid (**b**) sequences of the capsid proteins (ORF2) of the East African astrovirus field strains and the known astroviruses in the GenBank. Multiple sequence alignments were performed using the ClustalO program. The evolutionary history was inferred by using the maximum likelihood method on the GTR model for nucleotide and neighbor-joining method using the p-distance substitution model for amino acid sequences in MEGA X [33]. The scale bar is given in numbers of substitutions per site. Phylogeny was inferred following 1000 bootstrap replications, and the node values show percentage bootstrap support.

Genome sequence comparison revealed that the complete genomes of K456, K451, K366, and K062 strains shared relatively moderate to high nucleotide sequence identities among themselves (64.5 to 88.4%) and with the known sequences for PoAstV4 in the GenBank (63.5 to 80%), while low identities (41 to 48%) were noted with other AstV types (Table S1). The U083 and K321 strains had nucleotide sequence identities of 82% among themselves and 66.4 to 75.4% identity with the known sequences for PoAstV2 in the GenBank. U460 strain had nucleotide sequence identities of 57.0 to 65.5% with the known sequences for PoAstV3. These data demonstrate a wide genetic divergence among the PoAstV strains circulating in the East Africa region, and therefore, significant serological differences are expected because of the < 95% identity at the nt sequence level [11,46]. Further analysis of the nucleotide and deduced amino acid sequences of the capsid region revealed significant variation among the identified strains. Analysis of the nucleotide and deduced amino acid sequences of the capsid region revealed significant variations among East African field strains ranging from 51.7 to 77.8% and 49 to 76.4%, respectively, compared to other PoAstVs in the same group (Tables S2 and S3), suggesting that they are distinct/unusual PoAstVs within their respective genotypes; similar to reports has been documented in Japan [47]. We carried out phylogenetic analysis using the nucleotide and/or amino acid sequences of the complete genomes and capsid regions (ORF2) of the PoAstVs reported in this study and those available from GenBank, together with selected AstV sequences from other species to establish genetic relatedness (Figure 1 and Figure 2). In the phylogenetic trees constructed, East African strains clustered with astroviruses of the PoAstV2 (U083 and K321), PoAstV3 (U460), and PoAstV4 (K456, K451, K366, and K062) lineages.

The phylogenetic analysis of complete (nearly) genome showed our PoAstV2 strains were closely related at the nucleotide level, while the PoAstV4s were very diverse, consistent with our comparative sequence analysis results. The sequence identity between these novel viruses was generally greater in the RdRp region than in the NSP1a and capsid regions (data not shown). Capsid proteins are naturally under intense positive selective pressure from the host immune reaction [48]; hence they are likely to be more diverse, as shown in this study. According to the ICTV, the capsid protein encoded by the ORF2 is used to distinguish genotypes and species of astroviruses. They defined an amino acid sequence diversity in the capsid gene product of <0.312 and >0.378 within and between astrovirus species, respectively. Analysis of evolutionary divergence using the capsid region (ORF2) of our strains (Table S4) showed that PoAstV2 were 0.512 divergent among themselves and 0.418 to 0.579 with the known PoAstV2 strains in the same group. Similarly, PoAstV4 were diverse with 0.309–0.624 and 0.243–0.661 among themselves and with other known PoAstV4 strains, respectively. PoAstV3 was also diverse with 0.499–0.730 with known PoAstV3 strains. Thus, these results further confirm that our strains could be novel strains within their respective genotypes.

In this study, seven novel and genetically distinct PoAstVs have been described in fecal samples from apparently clinically healthy pig populations in East Africa. The phylogenetic analysis of nucleotides of complete genomes and ORF2 nucleotide and amino acid sequences of PoAstVs in our study revealed wider phylogenetic diversity than previously thought for the respective genotypes. These analyses confirmed that swine harbor phylogenetically diverse AstV strains, most likely derived from distinct ancestors, as has been report elsewhere [19].

### 3.3. Recombination Analysis

**Figure 3:**
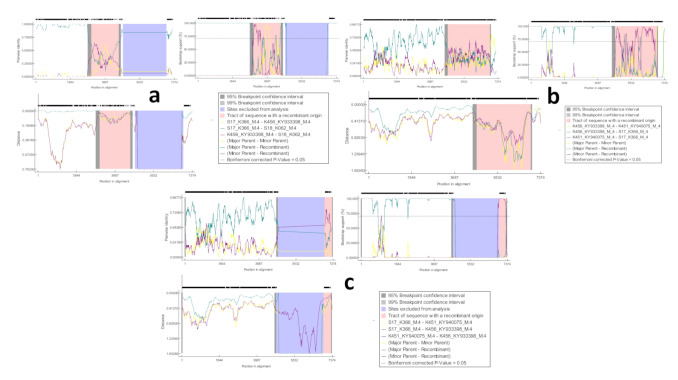
Recombination analysis of newly discovered full-length astrovirus genomes: (**a**) Predicted recombination event has likely occurred in the ORF1a–ORF1b junction and covers nearly the entire RdRp region. K062 strain is recombinant. (**b**) Predicted recombination event has likely occurred in RdRp–ORF2 overlap and covers almost the entire capsid region (ORF2); K366 is the recombinant. (**c**) Predicted recombination event has occurred in the 3′ end of the capsid region ORF2; K456 strain is recombinant.

Viral recombination can affect phylogenetic groupings, increase the virulence/fitness of the virus, complicate molecular epidemiological studies, and have major implications in vaccine design [49,50]. We further analyzed our strains for potential recombination, since our previous study identified multiple genotypes of PoAstV in same pigs and/or same farms [2,23]. The complete sequences of the seven new strains of astrovirus sequences were analyzed together with selected known AstV strains for probable recombination events. Between the four PoAstV4 strains (K456, K451, K366, and K062), three recombination events were identified: one starting at position 2713 and ending at position 4089 (event 1), another starting at position 4244 and ending at position 6305 (event 2), and the last event starting at position 6346 and ending at position 6649 (event 3) in K062, K366, and K456 respectively (Figure 3). Based on the recombination analysis, we concluded that all the four PoAstV4 strains may be of recombinant origin. Recombination event 1 (K062 recombinant) was predicted at the overlap between ORF1a and ORF1b, whereas recombination event 2 (K366 recombinant) was predicted from the junction of ORF1b-ORF2 and covered almost the entire ORF2. Event 3 (K456 recombinant) was predicted at the 3′ end of the capsid region (ORF2). These recombination patterns were supported by RDP, GENECONV, MaxChi, Chimaera, SiScan, 3Seq, LARD, Phylpro, and Bootscan programs, as shown in Table 2. Potential recombination event 2 reported in this study supports the previous suggestion that the ORF1b/ORF2 junction region is prone to the recombination region in AstVs [51,52]. The co-occurrence of different strains within swine farms is certainly feasible due to the high incidence of PoAstV in swine farms [2,43], and this may promote co-infections of individual pigs with multiple strains at the same time, as observed in this study. Therefore, recombination events between different strains have been observed. As an immune-escape mechanism, viral recombination events may lead to the generation of novel virus strains, to which the affected host may have a lower immunity than to the parent strains. These novel viruses may potentially cross species barriers at some point. Previous studies have found evidence for recombination amongst astrovirus serotypes in humans, pigs, marine mammals, turkeys, small ruminants, and dogs [51,53,54,55,56].

### 3.4. Prediction of Potential Linear Antigenic Epitopes

AstVs are not easy to propagate in cell culture; hence, comparisons of their antigenic properties are difficult. However, since the capsid protein induces host immunity, serological property is speculated based on its sequence similarity. Since AstV’s capsid protein is responsible for viral attachment and entry into host cells, we used capsid protein sequences of our strains to predict linear antigenic epitopes to generate information that could help in the design of vaccine components and immuno-diagnostic reagents. A total of 10 linear antigenic epitopes were identified in the capsid gene products of each of the seven field strains by SVMTrip Web-based software. The program-recommended antigenic linear epitopes predicted by SVMTrip tool for PoAstV4 strains were all antigenic in nature when analyzed by VaxiJen software; however, some of the recommended epitopes predicted by SVMTrip for PoAstV2 and PoAstV3 strains were non-antigenic in nature when analyzed by VaxiJen (Table S5). Therefore, to narrow down on the potential linear antigenic epitope candidates, which could be used as immunological targets, we further analyzed our sequences using IEDB analysis resource software and the immune-medicine group tool, which uses different algorithms to predict antigenic epitopes. The antigenic properties of identified target sequences from these epitopes were predicted based on threshold value of 0.4 by VaxiJen software. The sequences with values below the threshold value were considered non-antigenic, while sequences with values above threshold were considered antigenic in nature. Importantly, using the three analytic pieces of software above (SVMTrip, IEDB, and immune-medicine), among all the epitopes, we determined three potential candidate motifs at the surface of the structure that were present at same position on all the capsid proteins of PoAstV2, PoAstV3, and PoAstV4 strains, as shown in Table 3. The antigenicity of these predicted epitopes was further analyzed with VaxiJen software with a threshold of 0.4, which means the segments greater than the threshold were potentially antigenic in nature. Based on the results of the VaxiJen, we are proposing that the epitope at the amino acid position 126–161 is the best potential candidate epitope, since it contains a conserved motif in each genotype (Table 3).

**Figure 4:**
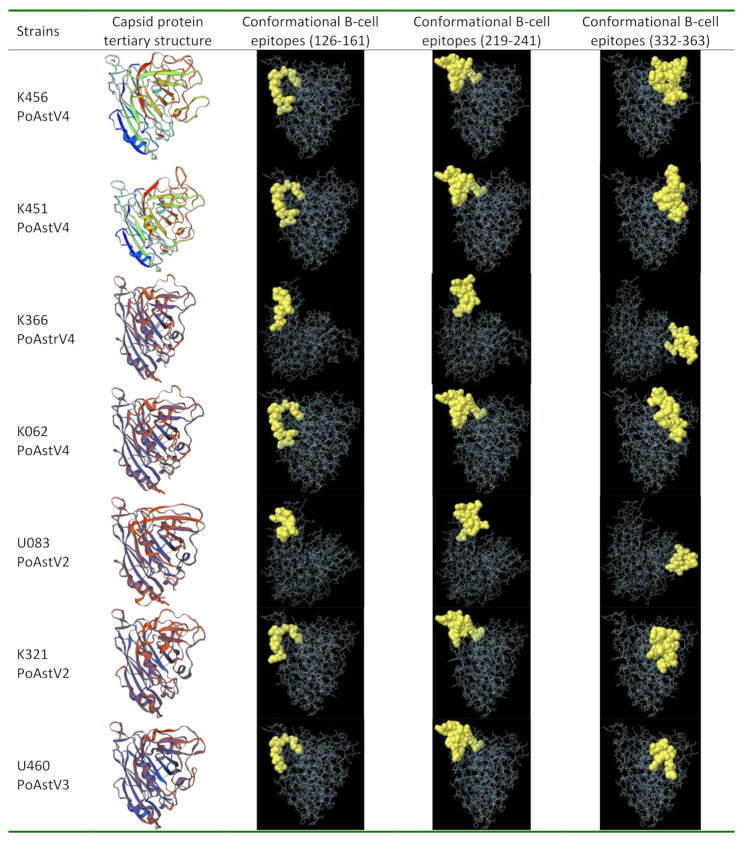
Capsid protein structures and conformational B-cell epitopes of PoAstV protein predicted by Ellipro from 3D structure template. Yellow balls are the residues of predicted peptides and white sticks are the non-epitope residues of protein. Each epitope is predicted with residue number and position mentioned.

After predicting the potential linear B cell epitope, we constructed 3D models of the capsid protein of each of our strains using Swiss model [40], to be able to predict potential conformational B-cell epitopes. We then predicted the conformational B-cell epitopes of our PoAstVs capsid protein models using Ellipro server [41]. The sequence identity between the capsid proteins of our PoAstV strains and the selected template (Q82452—human AstV 1 strain) ranged from 39.45 to 42.86%, which is more than the required 30% sequence similarity for generating useful models [57]. Our potential candidate epitopes predicted were identified in the capsid protein model by Ellipro software and visualized in Jmol to show their 3D structures or the relative orientations of protein and peptide molecules (Figure 4). The amino acid positions of each predicted epitope were also confirmed by Jmol viewer. The peptide was predicted to be highly antigenic (>1) and could be considered for effective immunization or immune diagnosis. Furthermore, all the selected epitopes were found at the surface of the structure, so could be used as the immunological targets for the proper diagnosis and treatment of PoAstVs in the study region. The generated information can be used to test the predicted functions of these peptides via in vitro and in vivo experiments.

### 3.5. Glycosylation Analysis

Glycosylation is generally required for progeny formation and infectivity of many viruses [58]. High levels of glycosylation of various pathogens serve as a protective shield from the host’s immune system, where during viral entry into host cells, glycans on the host cell represent viral receptors interacting with carbohydrate binding proteins on the viral surface [59,60]. We observed glycosylation on the capsid proteins of PoAstV2, PoAstV3, and PoAstV4, and more glycosylation sites in PoAstV2 compared to PoAstV3 and PoAstV4 (Table 4). Studies have shown that N- and O-linked glycans shield immunodominant epitopes from immune recognition [59,60,61]. Our analysis of the predicted antigenic epitopes at position 126**–**161 common between PoAstV2, PoAstV3, and PoAstV4 showed that they had at least one glycosylation site. Therefore, in-depth studies of the glycosylation in AstVs would be an important step in designing suitable antigens for diagnostic tools and vaccine development. Based on these results, we suggest that any approach that is based on inhibition of the host mechanism to glycosylate astrovirus proteins may offer the best potential approach to developing therapeutics for astrovirus infections.

## 4. Conclusions

To our knowledge, this is the first time that the complete genome sequence of a PoAstV from an African region has been determined and characterized. These data provide insights into the epidemiology and evolution of PoAstVs in the region and facilitate investigations on the genetic diversity of PoAstVs worldwide. The discovery of novel PoAstV strains described in this study provides an example of how diverse these viruses are in the smallholder pig population in the study region, where there is close contact between pigs and humans. Our data taken together would be helpful in the development of PoAstV immunodiagnostic tools and vaccines for African regions. However, it is important to note that the high genetic diversity among PoAstV strains (especially diversity at predicted potential antigenic epitopes) and high levels of glycosylation reported in the PoAstVs reported here may pose practical implications for virus detection methods, vaccine development, and epidemiological studies. Importantly, we have identified three potential linear antigenic epitopes which occurred at the surface of the structure of capsid protein, which could be used as the immunological targets for the proper diagnosis and treatment of PoAstVs in the study region. The generated information can be used to test the predicted functions of these peptides by conducting in vitro and in vivo experiments to confirm the immunogenicity and ultimately the vaccine properties to prevent PoAstV infections. Finally, understanding the genetic differences of these novel PoAstV variants that may emerge locally or globally through genetic drift or shift, in wild and domestic animals, could lead to early identification of the source of an emerging outbreak, leading to faster and more targeted interventions to control and/or limit the spread of such outbreaks. 

## Figures and Tables

**Table 1:** Comparison of the genomic organization of porcine astroviruses circulating in smallholder swine farms in East Africa.

Genotype	Strains	Accession No.	5′UTR	ORF1a	ORF1ab	ORF2	3′UTR	Total Length	Source
PoAstV2	U083	KY940077	18	2475	4053	2325	162	6434	Samia, Uganda
	K321	KY940076	27	2475	4056	2328	63	6347	Budalangi, Kenya
PoAstV3	U460 (Partial ORF1a)	KY933399	-	1616	3208	2148	-	5281	Budama, Uganda
PoAstV4	K456	KY933398	85	2550	3995	2511	67	6649	Funyula, Kenya
	K451	KY940075	-	2602	4138	2541	55	6634	Funyula, Kenya
	K366	MT451917	87	2550	3995	2469	76	6618	Funyula, Kenya
	K062	MT451918	87	2550	3995	2481	75	6629	Amukura, Kenya

(- = region missing).

**Table 2:** Summary of *p*-values of different recombination methods using the step-down correction for multiple comparisons between our field strains and a *p*-value cutoff of 0.01 in the Recombination Detection Program (RDP4, Version 4.94).

Method	Event 1 in K062	Event 2 in K366	Event 3 in K456
RDP	2.811 × 10^−13^	1.522 × 10^−13^	2.744 × 10^−16^
GENECONV	2.747 × 10^−17^	1.099 × 10^−2^	1.816 × 10^−3^
BootScan	2.199 × 10^−15^	4.858 × 10^−5^	1.518 × 10^−16^
MaxChi	2.377 × 10^−21^	9.780 × 10^−13^	4.161 × 10^−5^
Chimaera	2.805 × 10^−13^	2.604 × 10^−12^	5.516 × 10^−9^
Siscan	4.626 × 10^−2^	1.434 × 10^−7^	2.183 × 10^−13^
Phylpro	4.612 × 10^−9^	1.165 × 10^−14^	2.642 × 10^−13^
LARD	1.219 × 10^−71^	1.267 × 10^−76^	2.947 × 10^−22^
3Seq	4.612 × 10^−5^	1.165 × 10^−14^	2.642 × 10^−12^

**Table 3:** Predicted antigenic epitopes within capsid proteins (ORF2) of our field strains using three different pieces of software, and antigenicity of the predicted epitopes determined.

Strains	Amino Acid Position 126–161				Amino Acid Position 219–241			Amino Acid Position 332–363	
U083	FKMTKCELVLKPLVGDSAVSGTVVRASWNPTAT				IGKTMSTYQSRAFEGGLFLAELTT			RAANAPVRTGETTFDIYASISDARSDSPCVST	
K321	YKMTRCVVTLKPIVGDSAVAGTVTRVSWNPTSS				CHTFGKTTSTYRNEPFKGGLFLAE			VKRAAGAPVRANDNEIRFDIYASISDARSNTP	
U460	WRLTNLKIKCTPLVGPSAVTGSVYRVSLNLTQS				MIEIHGLGKTSSTYKDEPWVGDLF			PFQWLIKGGWWFVKKALGRSMNSDEVYYVYAS	
K456	WRVQYLDIKLTPLVGASAVSGTVIRTSLNLAAQ				TLGKTMSTYKSDIFDGPLFLAEVT			QWLIKAGWWFLKRIANKKKSGDHIDGQPDANE	
K451	WRVDNILIKLTPLVGASAVSGTAVRVSLNNAAT				TLGQTMSTYQAKVFTGPLFLCEMT			LFQAGWWFVKRIANKKKVGGSVDGEPDPGEVT	
K366	WRVKNMIIKLTPLVGGSAVSGTAVRTSLNLSGQ				TYGKTVSTYRNDPFTGPLFLAELT			LFKAGWWFVKKIANKSQNRNRPGEPDPGELTF	
K062	WRARDIIVKLTPLVGGSAVSGTAIRTSLNLSAQ				TLGKTLSTYKNEDFTGPLFLAELT			LFKAGWWFVKKIANKKTSGNAPGEPAPGELTF	
**Strains**		**SVMTriP** **Web-Based Tool**		**Immune-Medicine** **Group Tool**		**IEDB: Ellipro Software**		**VaxiJen Software**	
		**Position**	**Score**	**Position**	**Score**	**Position**	**Score**	**Position**	Antigenicity *
U083	PoAstV2	127–146	1.000	129–151	1.215	136–148	0.804	126–158	0.6357
K321		130–149	0.777	130–153	1.200	139–151	0.810	129–161	0.7207
U460	PoAstV3	-	-	135–161	1.150	142–154	0.809	132–164	1.0511
K456	PoAstV4	127–146	1.000	125–159	1.150	135–149	0.765	127–159	1.1882
K451		128–147	0.994	129–154	1.150	135–149	0.765	127–159	0.7063
K366		128–147	0.836	134–156	1.150	135–149	0.751	127–159	0.7977
Ko62		128–147	0.836	130–147	1.175	135–149	0.767	127–159	0.9112

* Antigenicity of the predicted epitope was analyzed by VaxiJen Software with threshold of 0.4 (values above threshold are probable antigens); a bold (red) amino acid sequence represents a motif conserved in each PoAstV type for positions 126–161 and 332–363, with high antigenicity (>1).

**Table 4:** Glycosylation analysis of the capsid proteins (ORF2) of astrovirus field strains using NetNGlyc software.

Name	Position	Sequence	Potential ^†^	Jury Agreement	N Glycosylated	Name	Position	Sequence	Potential ^†^	Jury Agreement	N Glycosylated
**U083**	12	NTTN	0.734	(9/9)	++	**U460**	160	NLTQ	0.750	(9/9)	+++
	20	NGSS	0.513	(5/9)	+		306	NATT	0.597	(6/9)	+
	55	NKTV	0.764	(9/9)	+++		625	NYTF	0.646	(8/9)	+
	86	NGSE	0.691	(9/9)	++	**K451**	274	NATP	0.110	(9/9)	---
	154	N**P**TA	0.697	(9/9)	++*		398	NITQ	0.681	(9/9)	++
	297	NKTI	0.752	(9/9)	+++		573	NYTM	0.731	(9/9)	++
	439	NYTT	0.648	(9/9)	++		658	NTTP	0.110	(9/9)	---
	542	NGTG	0.735	(9/9)	++	**K456**	251	N**P**TP	0.271	(8/9)	--
	557	NRTN	0.618	(7/9)	+		391	NITG	0.631	(9/9)	++
	611	NNTM	0.406	(8/9)	-		521	NPTL	0.643	(8/9)	+*
**K321**	13	NTTN	0.746	(9/9)	++		601	NGTL	0.698	(9/9)	++
	21	NGSS	0.505	(6/9)	+		655	NLTA	0.644	(9/9)	++
	41	NRTR	0.749	(9/9)	++	**K366**	123	NYSL	0.738	(9/9)	++
	56	NQSQ	0.560	(6/9)	+		155	NLSG	0.670	(9/9)	++
	80	NTTL	0.625	(9/9)	++		287	NGSL	0.573	(7/9)	+
	89	NESG	0.557	(6/9)	+		345	NKSQ	0.677	(8/9)	+
	157	N**P**TS	0.714	(9/9)	++*		502	NYTP	0.197	(9/9)	---
	412	NPTR	0.729	(9/9)	++*		559	N**P**TR	0.580	(8/9)	+*
	457	NGTK	0.685	(9/9)	++		565	NFTQ	0.584	(7/9)	+
	493	NNTT	0.547	(6/9)	+	**K062**	123	NYSL	0.709	(9/9)	++
	494	NTTA	0.609	(7/9)	+		155	NLSA	0.627	(8/9)	+
	511	NESP	0.129	(9/9)	---		287	NSSS	0.497	(4/9)	-
	560	NNSN	0.377	(9/9)	--		615	NQTV	0.607	(7/9)	+

^†^ = Any potential crossing the default threshold of 0.5 represents a predicted glycosylated site; + = N glycosylated; - = a negative site; * = proline occurs just after the asparagine residue, so is unlikely to be glycosylated; the jury agreement column indicates how many of the nine networks support the prediction; N = asparagine; S = serine; T = threonine. For picking up N-glycosylation sites with high specificity (asparagine residues very likely to be glycosylated), use only (++) predictions (and better) for asparagines that occur within the Asn–X–Ser/Thr triplet (no Proline at the X position).

## Supplementary Materials

The following are available online at https://www.mdpi.com/1999-4915/12/11/1262/s1. Table S1: Pairwise comparison of nucleotide sequence identities of the complete (near complete, U460) genomes of the seven (7) astrovirus field strains (bold) and with sequences of other astroviruses available in GenBank. Table S2: Summary of nucleotide sequence identity matrix of the capsid protein (ORF2) among the seven (7) astrovirus field strains (bold) and the known reference strains in the GenBank using Clustal Omega. Table S3: Summary of amino acid sequence identity matrix of the capsid protein (ORF2) among the 7 astrovirus field strains (bold) and the known reference strains in the GenBank using Clustal Omega. Table S4: Estimates of evolutionary divergence between the East African PoAstVs and selected known AstV in the GenBank based on the amino acid sequences of complete ORF2 protein. The number of amino acid differences per site from between sequences is shown. Standard error estimate(s) are shown above the diagonal for our strains. Table S5: Recommended potential linear antigenic epitopes predicted inside capsid protein (ORF2) of our field strains by SVMTriP Web-based tool and corresponding antigenicity predicted by VaxiJen software. Data Availability: The sequences of the strains discussed in this study have been deposited in GenBank under accession numbers KY940075-KY940077, KY933398, KY933399, MT451917, and MT451918. Additional data are presented in the supplementary files.
