# Supplementary material for: Whole Genome Sequence Analysis of Porcine Astroviruses Reveals Novel Genetically Diverse Strains Circulating in East African Smallholder Pig Farms
Source: Viruses. 2020 Nov 5;12(11):1262. doi: 10.3390/v12111262 (PMC7694451; doi:10.3390/v12111262)
Supplement: Supplementary file 1 [file viruses-12-01262-s001.pdf]

Supplementary Information

Table S1: Pairwise comparison of nucleotide sequence identities of the complete (near complete, U460) genomes of the seven (7) astrovirus field strains (**bold**) and with sequences of other astroviruses available in GenBank

**Strain name**	**U083**	**K321**	**U460**	**K456**	**K451**	**K366**	**K062**
**U083_KY940077_M.2**	100	81.86	44.09	46.93	47.36	46.99	46.64
**K321_KY940076_M.2**	81.86	100	44.7	47.62	47.11	47.62	47.39
PoAstV2_JPN_HgOg2-4_2015-LC201587	74.33	76.33	44.39	47.71	47.09	47.57	47.3
Astrovirus_2_KNU14-07	74.32	75.57	44.26	47.39	47.21	47.16	47.27
PAstV2_GER_L00855-K14_2014-LT898434	76.05	74.83	44.91	47.21	47.51	47.55	47.41
Astrovirus_2_Bel-12R021	73.11	74.3	43.73	47.04	47.53	47.32	47.58
PoAstV2_JPN_Ishi-Im3_2015-LC201593	71.36	74.04	43.56	48.03	47.22	47.91	47.67
AstV2-US-IA122	72.88	73.88	43.75	47.3	48	48.02	48.05
Camel_DcAstV-64	72.37	72.33	44.14	47.24	47.57	47.42	47.52
PoAstV2_Italy_DIAPD5469-10-MG930777	72.26	72.08	43.48	46.29	46.34	46.95	46.83
astrovirus_DcAstV-135	71.87	72.08	43.89	47.26	47.77	47.28	47.21
astrovirus_2_43_USA	71.29	72	44.2	47.35	47.7	48.01	48.37
Astrv2_ExpPig-36	71.19	72	44.00	47.67	48.19	48.29	48.19
M.3_AstV-LL-1-KP747573	71.47	71.56	44.05	47.09	47.4	47.15	46.92
PoAstV2_JPN_HgYa2-3_2015-LC201588	70.95	71.45	43.9	47.36	47.03	47.73	47.41
Camel_DcAstV-274	71.59	71.22	43.81	47.56	47.97	47.78	47.46
PoAstV2_BEL_15V010-KY214438	69.56	70.49	43.52	46.83	47.39	46.97	46.4
astrovirus_B76-2_HK	66.11	66.39	44.04	45.95	46.33	46.11	46.32
BAstV-GX27_CHN_2014	66.63	66.35	44.95	46.09	46.44	46.93	46.43
**K456_KY933398_M.4**	46.93	47.62	44.05	100	64.45	74.34	74.43
**K451_KY940075_M.4**	47.36	47.11	44.09	64.45	100	65.81	65.61
**S17_K366_ MT451917_M.4**	46.99	47.62	43.32	74.34	65.81	100	88.38
**S18_K062_ MT451918_M.4**	46.64	47.39	43.09	74.43	65.61	88.38	100
Astrovirus_4_15-14	47.14	47.6	43.21	73.86	63.34	74.55	74.89
AstV4-US-IL135	47.52	47.59	43.6	76.85	70.12	75.09	74.62
wild_boar_WBAstV-1-2011_HUN	46.05	47.13	43.16	76.48	65.78	78.9	76.74
WBAstV_CH_2015-KX033447	47.15	47.33	43.32	74.79	65.37	79.57	79.56
PoAstV4_JPN_Bu5-10-2_2014-LC201603	47.87	48.14	43.8	75.3	66.77	77.13	78.22
PoAstV4_CH_JXZS_2014-KX060809	47.31	47.17	43.32	77.46	64.65	75.19	75.69
PoAstV4_CH_JXJA-KX060808	47.44	47.63	43.06	75.31	66.72	77.14	77.27
Astrovirus_4_35_USA	47.98	48.04	43.51	74.39	69.96	75.74	75.63
PoAstV4_BEL_15V010-KY214437	47.15	47.48	42.71	72.67	63.57	73.73	74.38
PoAstV4_JPN_MoI2-1-1_2015-LC201609	47.79	48.06	43.69	76.77	64.96	74.63	73.36
PoAstV4_Tianjin_2018-MH425243	47.29	47.37	43.12	77.03	66	75.41	75.56
PoAstV4_JPN_HgTa2-3_2015-LC201608	47.38	47.72	43.48	72.22	65.34	73.27	73.77
**U460_KY933399_M.3**	44.09	44.7	100	44.05	44.09	43.32	43.09
Astrovirus_3_US-MO123	42.72	42.97	65.00	42.61	42.28	42.81	42.66
PoAstV3_USA_IA_7023_2017-KY940545	42.55	42.72	65.47	42.57	42.55	43.04	42.64
PoAstV3_JPN_Bu2-5_2014-LC201595	41.66	42.47	64.69	42.25	41.99	42.63	42.63
PoAstV3_JPN_Bu8-4_2014-LC201599	41.83	42.51	64.98	42.59	41.79	42.83	42.91
PoAstV3_NI-Brain_173-2016a_HUN-KY073231	41.86	42.41	65.00	42.34	42.41	43.2	43.23
PoAstv3_NI-Brain_9-2016a_HUN-KY073229	41.95	42.38	65.05	42.46	42.32	43.18	43.16
PAstV3_GER_L00919-K17_2014-KY073229	42.13	42.68	65.13	42.61	42.44	43.33	43.21
VA4_hu_Nepal_S5363_2008	41.53	42.07	56.73	43.01	41.66	43.12	43.14
Hu_astrovirus_BF34	41.97	42.41	57.31	42.42	42.06	42.22	42.2
Hu_VA1_FJ973620	41.97	42.55	55.55	42.52	41.91	42.89	42.61

Table S2. Summary of nucleotide sequence identity matrix of the capsid protein (ORF2) among the seven (7) astroviruses field strains (**bold**) and the known reference strains in the GenBank using Clustal Omega

Strain name	U083	K321	U460	K456	K451	K366	K062
**U083_KY940077_M.2**	100	61.48	36.34	41.33	42.27	41.2	40.66
**K321_KY940076_M.2**	61.48	100	34.93	42.51	40.28	42.78	42.77
2_astrovirus_2_KNU14-07	58.28	61.29	36.21	42.35	40.63	41.06	42.15
2_astrovirus_2_Bel-12R021	57.45	61.22	36.79	41.19	39.76	40.93	42.23
2_AstV2-US-IA122	62.76	64.82	36.74	40.52	42.37	42.35	42.34
2_astrovirus_2_43/USA	62.06	64.64	36.82	40.2	41.8	41.64	42.22
M.3/AstV-LL-1-KP747573	58.92	61.76	36.96	41.82	40.96	41.67	42.12
PoAstV2/JPN/HgOg2-4/2015-LC201587	57.77	62.72	36.94	41.33	40.9	41.31	41.01
PoAstV2/JPN/Ishi-Im3/2015-LC201593	57.7	65.01	35.73	41.73	40.2	42.6	41.9
PoAstV2/BEL/15V010-KY214438	54.43	55.17	35.16	39.89	40.26	40.24	39.61
PAstV2/GER/L00855-K14/2014-LT898434	68.23	62.62	37.34	40.89	41.74	41.45	42.11
PoAstV2/Italy/DIAPD5469-10-MG930777	60.6	58.09	36.1	40.36	39.89	40.93	41.46
PoAstV2/JPN/HgYa2-3/2015-LC201588	57.9	60.44	37.62	42.74	41.15	42.48	42.64
2_Astrv2_ExpPig-36	62.62	64.86	36.66	40.26	42.04	42.02	41.69
2_Camel_DcAstV-64	63.25	63.08	36.35	42.03	42.17	41.84	42.46
2_astrovirus_DcAstV-135	63.03	62.6	36.78	42.37	41.64	41.75	42.32
2_astrovirus_B76-2/HK	60.32	60.38	35.81	40.19	39.86	40.65	40.51
2_BAstV-GX27/CHN/2014	62.59	61.73	35.69	40.96	40.89	42.1	41.42
2-Camel_DcAstV-274	62.45	61.09	35.43	42.38	42.06	42.58	42
**K456_KY933398_M.4**	41.33	42.51	36.28	100	59.64	59.04	58.86
**K451_KY940075_M.4**	42.27	40.28	35.56	59.64	100	64.05	63.38
**K366_ MT451917_M.4**	41.2	42.78	33.79	59.04	64.05	100	74.92
**K062_ MT451918_M.4**	40.66	42.77	35.51	58.86	63.38	74.92	100
4_astrovirus_4_15-14	40.33	41.06	34.28	57.73	57.86	61.36	60.67
4_AstV4-US-IL135	41.69	41.51	35.82	61.88	75.44	62.26	60.45
4_wild_boar_WBAstV-1-2011_HUN	40.53	42.54	35.1	61.22	65.67	77.81	72.37
WBAstV/CH/2015-KX033447	40.93	42.07	36.45	61.52	61.88	69.91	68.68
PoAstV4/JPN/Bu5-10-2/2014-LC201603	41.15	42.92	34.74	60.34	65.92	65.06	66.65
PoAstV4/CH/JXZS/2014-KX060809	41.87	41.5	35.09	69.43	60.64	59.08	59.28
PoAstV4/CH/JXJA-KX060808	41.01	41.48	35.12	60.9	66.08	64.43	63.71
4_astrovirus_4_35_USA	41.96	41.46	35.46	60.98	75.16	61.57	59.77
P._astrovirus_4/BEL/15V010-KY214437	41.43	41.56	35.57	59.94	58.04	61.07	61.01
PoAstV4/JPN/MoI2-1-1/2015-LC201609	42.03	42.9	35.33	72.62	60.57	59.5	57.98
PoAstV4/Tianjin/2018-MH425243	41.64	42.34	36.27	58.83	61.62	63.99	64.51
PoAstV4/JPN/HgTa2-3/2015-LC201608	41.95	42.12	36.68	60.31	61.66	62.06	62.18
**U460_KY933399_M.3**	36.34	34.93	100	36.28	35.56	33.79	35.51
3_astrovirus_3_US-MO123	33.89	36.55	59.34	35.51	37.03	35.58	36.94
PoAstV3/USA/IA/7023/2017-KY940545	33.72	36.14	59.15	35.36	37.37	35.56	36.61
PoAstV3/JPN/Bu2-5/2014-LC201595	34.05	36.56	58.96	34.87	36.71	34.79	36.14
PoAstV3/JPN/Bu8-4/2014-LC201599	33.52	35.78	59.11	35.2	36.14	35.12	35.69
PoAstV3/NI-Brain/173-2016a/HUN-KY073231	33.37	35.29	59.24	35.45	37.38	34.81	35.37
PoAstv3/NI-Brain/9-2016a/HUN-KY073229	33.52	35.29	59.19	35.54	37.01	34.81	35.28
PAstV3_GER_L00919-K17_2014-KY073229	34.33	36.35	59.71	35.14	36.65	36.09	37.11
3_VA4_hu_Nepal_S5363_2008	36.77	37.11	51.71	36.68	36.5	36.17	37.52
3_Hu_astrovirus_BF34	36.99	37.29	52.66	36.19	36.33	34.99	35.42

Table S3. Summary of amino acid sequence identity matrix of the capsid protein (ORF2) among the 7 astroviruses field strains (**bold**) and the known reference strains in the GenBank using Clustal Omega

Strain name	U083	K321	U460	K456	K451	K366	K062
**U083_KY940077_M.2**	100	58.45	21.4	31.38	29.96	28.44	30.01
**K321_KY940076_M.2**	58.45	100	22.52	31.38	28.7	30.79	31.82
P.Astrv2_KNU1407	54.99	58.96	22.12	29.91	31.26	31.02	31.66
P.AstrV2_PFTS-2	59.95	65.55	22.24	31.6	30.96	31.23	32.47
AstV2USIA122	60.35	65.68	22.08	31.74	30.82	30.82	31.79
PoAstV2/JPN/HgOg2-4/2015	56.08	63.78	22.55	30.36	30.23	31.41	31.11
D.AstrV_DcAstV-135	60.26	60.32	22.71	32.44	29.84	29.38	30.29
P.AstrV2_GXBS5/2013	59.39	64.85	20.34	30.11	29.3	29.4	29.92
B.AstrV_BAstGX-J8	59.22	60.89	22.26	31.02	29.23	30.38	31.02
P.Astrv2_ExpPig36	60.3	65.73	22.43	32.1	31.32	30.22	32.28
Camel_DcAstV64	59.6	59.65	21.46	32.31	29.57	29.78	30.97
P.AstrV2_51/USA	61.16	63.32	22.02	32.75	30.24	31.36	31.99
A.swine/PoAstV12-4/Canada/2006	59.45	76.4	22.46	31.49	29.48	31.31	30.87
B.Astrv_B762/HK	53.94	56.24	21.58	30.25	28.38	29.88	30.71
**U460_KY933399_M.3**	21.4	22.52	100	22.59	22.53	22.49	23.03
P.Astrv3_USMO123	21.17	20.9	59.32	22.35	21.23	20.88	21.31
PoAstV162/Canada/2006	20.87	20.9	59.04	22.21	20.79	20.88	21.46
H.Astrovirus_BF34	21.08	21.24	48.9	23.03	23.68	23.02	23.9
VA4/human/Nepal/S5363/2008	21.02	22.24	48.75	21.88	21.35	22.04	22.63
**K456_KY933398_M.4**	31.38	31.38	22.59	100	54.21	55.71	54.21
**K451_KY940075_M.4**	29.96	28.7	22.53	54.21	100	60.57	59.61
**K366_ MT451917_M.4**	28.44	30.79	22.49	55.71	60.57	100	73.29
**K062_ MT451918_M.4**	30.01	31.82	23.03	54.21	59.61	73.29	100
PoAstV4/JPN/Ishi-Ya7-1/2015	31.28	31.14	21.52	65.38	54.78	53.45	52.33
PoAst4/US-P2011-1	32	30.12	20.69	64.27	56.5	54.72	53.48
PoAst4/JXJA/CHINA	29.6	30.08	22.49	55.54	63.41	61.04	60.37
PoAst4/USA/15-12	30.4	29.85	21.67	51.95	53.32	57.16	56.72
WBAstV/CH/2015	27.81	29.49	21.48	56.55	58.43	66.22	66.46
PoAstrv4_35/USA	30.84	29.18	23.07	53.69	73.93	55.17	53.56

Table S4: Estimates of evolutionary divergence between the East African PoAstVs and selected known AstV in the GenBank based on the amino acid sequences of complete ORF2 protein. The number of amino acid differences per site from between sequences is shown. Standard error estimate(s) are shown above the diagonal for our strains.

Strain name	K456	K451	K0366	K062	U083	K321	U460
**K456_KY933398_M.4**		0.032	0.031	0.032	0.053	0.051	0.071
**K451_KY940075_M.4**	0.624		0.029	0.028	0.055	0.055	0.075
**K366_ MT451917_M.4**	0.585	0.509		0.022	0.056	0.057	0.071
**K062_ MT451918_M.4**	0.620	0.522	0.309		0.055	0.055	0.070
**U083_KY940077_M.2**	1.165	1.234	1.255	1.210		0.029	0.071
**K321_KY940076_M.2**	1.172	1.276	1.226	1.178	0.512		0.070
**U460_KY933399_M.3**	1.534	1.510	1.553	1.532	1.538	1.550	
P.Astrv2_KNU1407	1.226	1.191	1.198	1.189	0.571	0.516	1.535
P.AstrV2_PFTS-2	1.182	1.182	1.191	1.152	0.484	0.419	1.536
AstV2USIA122	1.170	1.183	1.188	1.154	0.475	0.429	1.547
PoAstV2/JPN/HgOg2-4/2015	1.231	1.228	1.183	1.202	0.568	0.446	1.499
D.AstrV_DcAstV-135	1.168	1.224	1.229	1.198	0.473	0.488	1.481
P.AstrV2_GXBS5/2013	1.223	1.266	1.258	1.225	0.516	0.425	1.596
B.AstrV_BAstGX-J8	1.178	1.237	1.202	1.185	0.487	0.487	1.553
P.Astrv2_ExpPig36	1.179	1.175	1.211	1.154	0.475	0.418	1.534
Camel_DcAstV64	1.164	1.233	1.224	1.185	0.491	0.504	1.501
P.AstrV2_51/USA	1.127	1.214	1.182	1.167	0.480	0.455	1.543
A.swine/PoAstV12-4/Canada/2006	1.188	1.252	1.207	1.222	0.502	0.261	1.517
B.Astrv_B762/HK	1.237	1.289	1.238	1.221	0.579	0.556	1.527
P.Astrv3_USMO123	1.565	1.604	1.642	1.604	1.589	1.630	0.499
PoAstV162/Canada/2006	1.586	1.611	1.649	1.604	1.604	1.645	0.503
H.Astrovirus_BF34	1.536	1.517	1.482	1.437	1.627	1.577	0.711
VA4/human/Nepal/S5363/2008	1.599	1.601	1.591	1.554	1.630	1.524	0.730
wild_boar/WBAstV1/2011/HUN	0.562	0.479	0.243	0.361	1.243	1.222	1.579
PoAstV4/JPN/MoI2-1-1/2015	0.330	0.595	0.612	0.645	1.156	1.180	1.604
PoAstV4/CH/JXZS/2014	0.391	0.557	0.584	0.589	1.163	1.183	1.578
PoAstV4/JPN/Ishi-Ya7-1/2015	0.429	0.626	0.633	0.661	1.165	1.202	1.578
PoAst4/US-P2011-1	0.443	0.591	0.606	0.630	1.165	1.216	1.620
PoAst4/JXJA/CHINA	0.583	0.446	0.493	0.501	1.214	1.232	1.536
PoAst4/USA/15-12	0.662	0.643	0.559	0.565	1.222	1.257	1.557
WBAstV/CH/2015	0.567	0.544	0.405	0.403	1.268	1.244	1.589
PoAstrv4_35/USA	0.620	0.291	0.589	0.619	1.198	1.247	1.494

Table S5. Recommended potential linear antigenic epitopes predicted inside capsid protein (ORF2) of our field strains by SVMTriP web-based tool and corresponding antigenicity predicted by VaxiJen software

Genotype	Name	Rank	Location	Epitope	Score	Recommended by SVMTriP tool	Overall Prediction for the Protective Antigen (VaxiJen)	Probability of being antigen (VaxiJen)
PoAstV 4	K456-S1	1	127 - 146	*WRVQYLDIKLTPLVGASAVS	1.000	🚩	1.6170	Antigen
		2	662 - 681	VTLSMVHQLVQQALAERNNQ	0.619		0.6085	Antigen
		3	67 - 86	KSSNSMYSQRITATLGSVGA	0.591		0.8726	Antigen
		4	765 - 784	IKNLLTPEAKDLYGDLRRKG	0.569		0.4459	Antigen
	K451-S7	1	561 - 580	SPTTLPSFIETIKITDTISV	1.000	🚩	0.5850	Antigen
		2	128 - 147	*RVDNILIKLTPLVGASAVSG	0.994	🚩	0.7819	Antigen
		3	509 - 528	MVYAASHTAIGSNPTLHWTT	0.936	🚩	0.5821	Antigen
	K366-S17	1	651 - 670	APTALTTREILALREMMNGQ	1.000	🚩	0.6738	Antigen
		2	514 - 533	YARIDNGPLHWTTCLWRATV	0.992	🚩	0.4613	Antigen
		3	128 - 147	*RVKNMIIKLTPLVGGSAVSG	0.836	🚩	0.6293	Antigen
	K062-S18	1	651 - 670	APTALTTREILALREMMNGQ	1.000	🚩	0.6738	Antigen
		2	514 - 533	YARIDNGPLHWTTCLWRATV	0.992	🚩	0.4613	Antigen
		3	128 - 147	*RVKNMIIKLTPLVGGSAVSG	0.836	🚩	0.6293	Antigen
PoAstV2	U083-S5	1	127 - 146	*KMTKCELVLKPLVGDSAVSG	1.000	🚩	0.1348	Non-Antigen
		2	743 - 762	SDEYTEFTEVYHDALADGLS	0.962	🚩	0.0943	Non-Antigen
		3	267 - 286	ARLKTDENGKMQLVLPTSSS	0.713		0.0592	Non-Antigen
		4	87 - 106	GSEQIECEMTCLLNPATMKE	0.687		0.7600	Antigen
		5	591 - 610	IIASRGTEAVVRGDHYFTPS	0.684		0.6845	Antigen
		6	703 - 722	YSDPPISRLVVRDDAVALYE	0.650		0.2461	Non-Antigen
		7	657 - 676	FGFDDASEFPPPPSEEDPAE	0.608		0.5654	Antigen
	K321-S13	1	454 - 473	VGFVCNGTKVCSFTVHKVEV	1.000	🚩	0.6249	Antigen
		2	93 - 112	SGEIECELTCLMNPATMKEV	0.850	🚩	0.8868	Antigen
		3	130 - 149	*LYKMTRCVVTLKPIVGDSAV	0.777		-0.0338	Non-Antigen
		4	286 - 305	LPRNSRMARAATTTPSEIIW	0.612		0.2482	Non-Antigen
		5	496 - 515	NTTANLGNAVASAYVKANES	0.527		0.5669	Antigen
		6	63 - 82	RRRVLRRQGVGDRVVFQKIN	0.514		-0.4204	Non-Antigen
PoAstV3	U460-S9	1	578 - 597	TSQAPTTINADKIAIPKMAT	1.000	🚩	-0.2183	Non-Antigen
		2	345 - 364	KKALGRSMNSDEVYYVYASL	0.967	🚩	0.1499	Non-Antigen
		3	306 - 325	NATTVGETIWQIVDEGANLV	0.822	🚩	0.5468	Antigen
		4	680 - 699	RQTSDHVDAVIQEIQRRFRL	0.804	🚩	0.5556	Antigen

* The epitopes common to all genotypes except PoAstV3. Analysis carried out with web based Support Vector Machine software which combines the Tri-peptide similarity and Propensity scores (SVMTriP) in order to achieve the better prediction performance (Yao et al. 2012). While antigenicity of predicted epitopes was determined using VaxiJen v2.0 protective antigen, tumour antigens and subunit vaccines prediction server (Doytchinova and Flower, 2007).

Data availability: The sequences of the strains discussed in this study have been deposited in GenBank under accession numbers KY940075-KY940077, KY933398, KY933399, MT451917, and MT451918. Additional data are presented in the supplementary files.
